# Preventing What Matters: A Fast and Reliable Technique to Secure External Ventricular Drains and Avoid Dislodgement

**DOI:** 10.7759/cureus.85711

**Published:** 2025-06-10

**Authors:** Sevgi Sarikaya-Seiwert, Christian Wispel, Muriel Heimann, Sarah-Marie Gallert, Hartmut Vatter, Ehab Shabo

**Affiliations:** 1 Section of Pediatric Neurosurgery, Department of Neurosurgery, University Hospital Bonn, Bonn, DEU; 2 Department of Neurosurgery, University Hospital Bonn, Bonn, DEU

**Keywords:** catheter dislodgement, evd fixation, evd-related morbidity, external ventricular drain, hydrocephalus

## Abstract

Background

External ventricular drain (EVD) placement is a critical neurosurgical procedure for managing intracranial pressure and represents the most performed procedure in neurosurgery. However, complications such as dislodgement and accidental removal remain prevalent, often resulting in possible significant morbidities. While various fixation techniques exist, a standardized method to minimize these risks is lacking and remains institution-dependent. This study aims to introduce and evaluate a novel EVD fixation technique developed from clinical experience, aiming to reduce complications associated with EVD dislodgement and accidental removal.​

Methods

In this retrospective study, we evaluate a new suturing method for EVD fixation in all pediatric patients treated at our institution between 2020 and 2024 who underwent an EVD implantation due to various indications (n=62). All EVDs were placed at Kocher’s point. The two-step fixation technique was documented with detailed step-by-step photographs and was applied consistently across the cohort. All patients were monitored for EVD-related complications, including dislodgement, leakage, infection, and malfunction.

Results

Sixty-two pediatric patients were included in this study. Indications for EVD included posterior fossa tumor surgery (n=39), shunt infection and intracerebral or intraventricular hemorrhage (n=22), and intracerebral abscess (n=1). In three of 62 patients, the secondary fixation suture was inadvertently cut during EVD removal, requiring bedside re-suturing of the EVD exit point. In one patient, the primary fixation suture was applied too tightly, leading to reduced cerebrospinal fluid drainage and necessitating bedside re-fixation of the EVD. Importantly, no cases of EVD dislodgement, migration, leakage, or infection were observed in this cohort.

Conclusion

The presented EVD fixation technique demonstrated both safety and efficacy, providing excellent catheter stability with no observed cases of dislodgement or leakage. Notably, the method obviates the need for additional suturing of the exit point following EVD removal, thereby reducing procedural discomfort and avoiding unnecessary pain, which is particularly relevant in the pediatric population. These findings indicate that this technique may substantially lower the incidence of EVD-related complications and has the potential to serve as a standardized fixation approach. Further prospective studies with larger cohorts are needed to validate these preliminary results.

## Introduction

External ventricular drains (EVDs) are essential tools in neurosurgery for monitoring and managing intracranial pressure and represent the most frequently performed procedures in the field [1-5].

Despite their widespread use, EVDs are prone to complications, particularly dislodgement and accidental removal, which can result in elevated intracranial pressure, intraventricular hemorrhage, infection, and the need for additional surgical interventions. Various fixation techniques have been proposed to secure EVDs and therefore mitigate these risks, including the use of sutures, staples, and adhesive devices.

For example, Velásquez et al. described a fixation method using hydrocolloid dressings and staples, achieving a low pullout rate of 0.4% [2]. In contrast, Salem et al. conducted a 10-year retrospective review and reported a dislodgement rate of 5.9%, with no significant difference between staples and sutures as fixation methods [6]. Akarca et al. presented a comprehensive evaluation of 15 commonly used EVD fixation techniques by combining survey data from 23 UK neurosurgical centers with a biomechanical assessment in a porcine cadaver model. Their findings identified a triple-fixation strategy, consisting of an anchoring suture, a coiled suture around the catheter, and a soft or hard plastic flange, as the most secure, withstanding peak pull forces up to 29.05 N [4]. However, the study was experimental in nature, with a focus on measuring the mechanical strength of various EVD securement techniques by assessing the peak pull force required to cause catheter failure, and did not report clinical dislodgement rates. Shimizu et al. proposed a novel fixation technique to address mechanical EVD obstruction in patients undergoing long-term frontal horn placement. Using a catheter fixation system comprising a dialysis catheter wing and a fixture spring, their method allowed controlled retraction of the EVD to restore flow. In their cohort consisting of 20 patients, three EVD-related complications were reported: two cases (10%) of subarachnoid hemorrhage with diffuse brain swelling and one case (5%) of insertion-site infection [7]. Daniel et al. introduced a specialized cranial fixation device designed to improve EVD accuracy and security in patients with thin skulls, such as neonates. The device demonstrated accurate ventricular cannulation and strong fixation in both ex vivo and in vivo animal models, withstanding pullout forces of up to 4.18 kgf [8]. Nevertheless, the study focused on calculating pullout forces and did not report dislodgement or complication rates. Moreover, the requirement for an additional specialized device limits its universal applicability.

In a low-resource context, Bhandari et al. evaluated a cost-effective fixation method involving coiling the EVD catheter around a soft plastic flange and securing it with sutures. In their retrospective review of 107 cases, only one deliberate self-removal of EVD was reported (0.93%), with no accidental dislodgements [9]. This technique offered a practical, low-cost solution, especially in settings where commercial devices are unavailable. However, the study lacked assessment of other potential complications, such as long-term fixation stability or infection, and did not fully address the method's resilience in agitated or mobile patients.

Despite the variety of methods explored, a universally accepted standard for EVD fixation has yet to be established.

Given the persistent challenges associated with EVD fixation, we developed a novel suturing technique aimed at improving catheter stability and eliminating the risk of dislodgement without the need for additional tools or devices, making it particularly suitable for diverse healthcare environments, including those with limited resources.

## Materials and methods

Patient selection and data collection

This retrospective study included all pediatric patients who underwent external ventricular drain (EVD) placement at our institution between January 2020 and December 2024.

Inclusion criteria were patients under 18 years of age at the time of the procedure, and all cases were included regardless of the underlying indication for EVD insertion. Common indications included posterior fossa tumor surgery (n=39), shunt infection with a history of intracranial or intraventricular hemorrhage (n=22), and intracerebral abscess (n=1).

Patients were excluded if they were 18 years of age or older at the time of EVD placement, if the procedure occurred outside the defined study period, or if relevant clinical data were incomplete or unavailable in the medical records.

Clinical data were collected retrospectively from electronic medical records, including patient demographics and indications for EVD placement, with a primary focus on EVD-related complications, particularly dislodgement, accidental removal, and cerebrospinal fluid (CSF) leakage.

Surgical workflow

The described novel EVD fixation technique was originally developed and refined over more than a decade by one of the senior pediatric neurosurgeons on the team, who had used it independently in clinical practice. In 2020, this technique was formally adopted as the standard fixation method within our pediatric neurosurgery unit, which comprises three neurosurgeons: the senior developer of the technique, a newly board-certified neurosurgeon with no independent experience beyond residency, and a fourth-year neurosurgery resident. Following a single instructional demonstration by the senior neurosurgeon, both the junior attending and the resident successfully applied the technique under supervision. Thereafter, they were able to perform the fixation independently and without difficulty, indicating the technique’s ease of adoption and reproducibility across varying levels of surgical experience.

All procedures were performed by the pediatric neurosurgery team under sterile conditions in the operating room with the patient under general anesthesia.

For EVD placement, patients were positioned supine with the head in a neutral position, slightly elevated at approximately 30°. Kocher’s point was identified using anatomical landmarks, 2-3 cm paramedian just anterior to the coronal suture. This location corresponds to the mid-pupillary line in the coronal plane and ensures entry into the frontal horn of the lateral ventricle, avoiding the motor cortex. A C-form skin incision of approximately 2-3 cm was made over the marked Kocher’s point. Blunt dissection was carried down to the pericranium, which was incised and reflected. A burr hole was created using a high-speed drill. Hemostasis was secured using bone wax as needed. The dura was coagulated and incised in a cruciate fashion. A small corticotomy was made using bipolar cautery and a fine scalpel. A standard external ventricular catheter was then inserted perpendicular to the cortical surface and advanced approximately 5-6 cm toward the ipsilateral medial canthus, following the trajectory toward the foramen of Monro. In most cases, ultrasound navigation was used to place the EVD catheter. Free cerebrospinal fluid (CSF) flow confirmed intraventricular placement.

Once correct intraventricular placement of the catheter was confirmed by free cerebrospinal fluid outflow, the EVD was tunneled dorsally through the subcutaneous tissue using a sharp tunneler. To avoid infections, the exit of the drain was at least 4 to 5 cm away from the incision. Fixation was then performed using a standardized two-step suture technique specifically designed to enhance catheter stability, minimize the risk of dislodgement, and avoid additional suturing after EVD removal.

Description of the EVD fixation technique and further course

**Figure 1:**
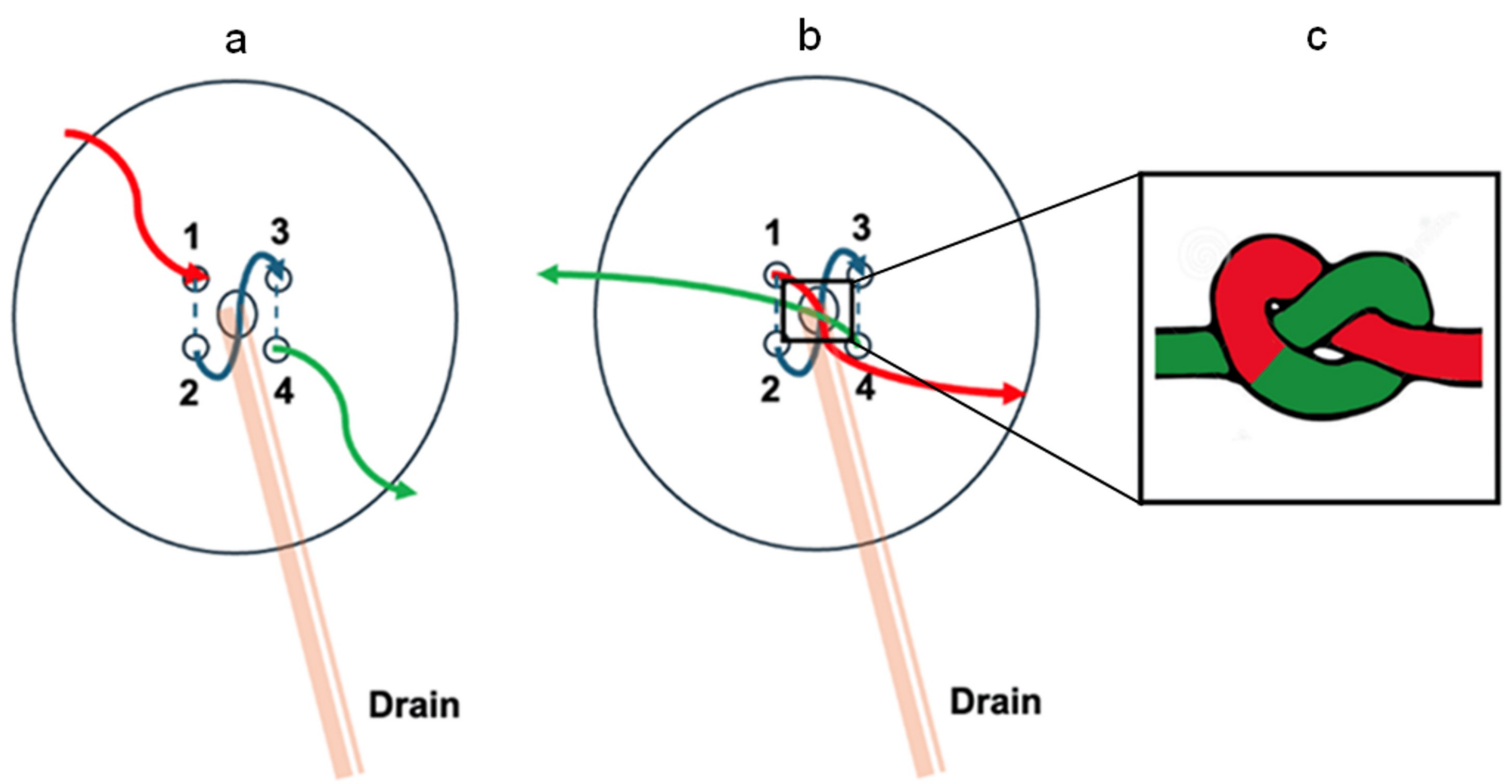
Illustration of the first step of a two-step-suture fixation technique for securing an external ventricular drain (EVD) at its exit point The image shows a top-down schematic view of the scalp around the EVD exit point. The orange structure labeled “Drain” represents the EVD exiting the scalp. (a) The colored lines represent the direction of the fixation suture, which is applied in an inverse N-shaped configuration to anchor the drain securely to the skin. The needle enters at point 1 (red line anterior to the exit point) and exits at point 2 (blue line posterior). It then enters again at point 3 (anterior on the other side) and exits at point 4 (green line posterior on the other side). The subcutaneous part of the suture is parallel to the EVD. (b) The loose ends of the suture are knotted primarily over the EVD as demonstrated in (c). Figure 1 was created by author Dr. Sarikaya-Seiwert, using Microsoft PowerPoint, 16.97 (Microsoft Corporation, Redmond, WA, US); used with permission.

**Figure 2:**
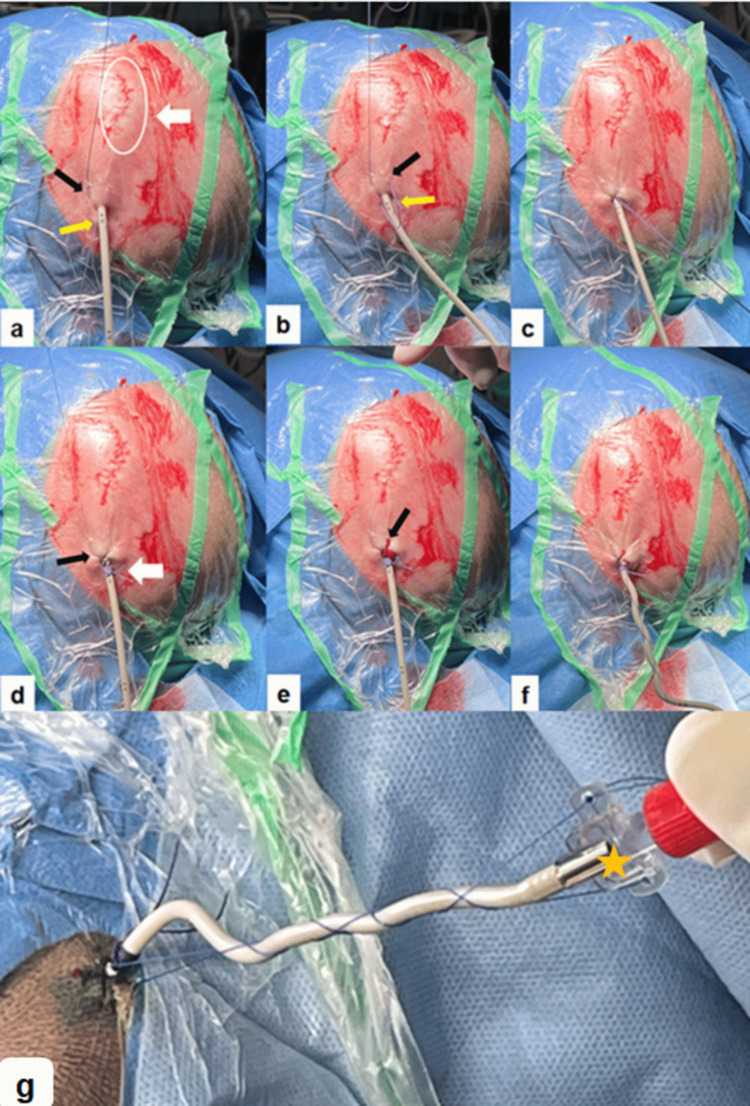
The step-by-step intraoperative application of the two-step-suture fixation technique for securing the EVD This image sequence (a–g) illustrates the two-step suture technique: (a) The scalp is prepped and draped for sterile EVD placement. The white circle and white arrow mark the C-shaped skin incision over Kocher’s point on the left side. The black arrow points to the initial entering point of the first absorbable suture, anterior and lateral to the EVD exit point. The suture exits caudally (yellow arrow). (b) The second needle pass is performed in a mirrored fashion on the right side of the drain, from the entry point (black arrow) to the exit point (yellow arrow), creating the characteristic N-shape around the EVD. (c) The first suture is knotted primarily. (d) The suture is now wrapped multiple times around the EVD, creating a friction-locking effect that securely anchors the catheter to the scalp (white arrow). The black arrow points to the entering point of the second non-absorbable suture laterally to the entering point of the first suture. (e) The same technique is used to perform the second needle pass of the second non-absorbable filament. The black arrow points to the entrance point. (f) The second suture is wrapped, relaxed around the EVD toward its distal end. (g) The final appearance of the EVD after the two-step fixation technique is completed. Both ends of the second suture are passed through the holes of the wing-shaped connector (orange star) and then knotted in a way that makes sure that the relaxed suture is 2 to 3 cm shorter than the length of the visible catheter. The drain is securely anchored, with no extra anchoring devices required. The second suture that is wrapped around the EVD to its distal end will also serve a second purpose: after EVD removal, it can be tightened to close the EVD exit site, eliminating the need for additional suturing and minimizing patient discomfort. EVD: external ventricular drain

In the first step, an absorbable suture (Vicryl®) is passed through the cutis parallel to the EVD on one side in a craniocaudal direction and repeated on the other side, forming an N-shaped or inverse-N-shaped (zigzag) configuration (Figures 1, 2). After making a knot over the drain, the suture was then wrapped circumferentially around the catheter several times and securely knotted to provide primary stabilization (Figure 2).

In the second step, a non-absorbable suture (Prolene®) is applied using the same directions and technique as in the first step and slightly laterally from it (Figure 2). This second suture is wrapped, relaxed around the catheter, and both ends are passed through the holes of the wing-shaped connector piece at the distal end of the EVD catheter. Behind the connector, a final knot is tied with the relaxed ends of the suture, making sure that the relaxed suture is 2 to 3 cm shorter than the length of the visible catheter, generating a counterforce mechanism that effectively resists any axial traction or accidental pull-outs. The distal end is then connected to a closed sterile external drainage system. Both sutures are performed in a parallel direction to the EVD to minimize the risk of accidental puncture of the EVD. The C-form skin incision at Kocher’s point is closed using an absorbable suture for the subcutis and cutis (Figure 2).

A few days after the surgery, and depending on the patient’s EVD dependency, the EVD is removed bedside in the pediatric neurosurgical ward. To remove the EVD, the second, non-absorbable suture is cut distally near the connector to loosen both suture ends and free them from the EVD, then the first absorbable suture is cut proximally near the skin to loosen the EVD. Hereafter, the EVD is removed, and the second, non-absorbable suture is directly knotted to close the exit point of the removed EVD.

## Results

A total of 62 pediatric patients underwent EVD placement using the described two-step fixation technique at Kocher’s point during the study period between January 2020 and December 2024. The mean age at the time of EVD insertion was 7 years (range: 1-16). The cohort was nearly evenly distributed by sex, with 27 females (43.5%) and 35 males (56.4%) (Table 1).

**Table 1:** Detailed patient characteristics CSF: cerebrospinal fluid; EVD: external ventricle drainage; ICH: intracerebral or hemorrhage; IVH: intraventricular hemorrhage; No.: number

No. of Patients		62
Age at EVD (years)		mean: 7 (range: 1-16)
Sex	Female	27 (43.6%)
	Male	35 (56.4%)
EVD indication	Posterior fossa tumor surgery	39 (62.9%)
	Shunt infection with a history of ICH or IVH	22 (35.5%)
	Intracerebral abscess	1 (1.6%)
EVD type	Silicone	24 (38.7%)
	Silverline	38 (61.3%)
EVD-related complications	Dislodgement	0
	CSF leakage	0
	Infection	0
	Overtightened suture	1 (1.6%)
	Accidental cut of the second suture	3 (4.8%)
	EVD revision	0
Days to remove the EVD (days)		median: 8 (q1-q3: 5-12)

An indication for EVD insertion was posterior fossa tumor surgery, accounting for 39 cases (63%). Additional indications included shunt infection in the context of a history of intracerebral (ICH) or intraventricular hemorrhage (IVH) in 22 patients (35.5%) and intracerebral abscess in one patient (1.6%).

Regarding the surgical approach, Kocher’s point was used as described in the methods. A silicone EVD was used in 24 patients (38.7%), and a Silverline EVD (Spiegelberg GmbH & Co. KG, Hamburg, Germany) was used in 38 patients (61.3%).

The median duration until EVD removal was 8 days, with an interquartile range of q1-q3: 5-12.

Regarding procedural outcomes and complications, the described two-step fixation technique demonstrated a high degree of safety and effectiveness. In 3 out of 62 patients (4.8%), the second non-absorbable fixation suture was inadvertently cut during EVD removal, necessitating simple bedside re-suturing of the catheter exit point. In one patient (1.6%), the first absorbable fixation suture had been applied with excessive tension, resulting in impaired cerebrospinal fluid (CSF) drainage. This issue was promptly identified postoperatively and resolved with a bedside re-fixation in the pediatric ward.

Crucially, no cases of EVD dislodgement, dislocation, migration, or infection were observed throughout the postoperative monitoring period, and no patient required EVD revision.

The overall complication rate directly attributable to the fixation technique was 6.5%, all of which were minor, easily correctable, and did not result in long-term sequelae.

## Discussion

The present study demonstrates that the described two-step suturing technique for EVD fixation is a highly effective and practical method for preventing catheter dislodgement and accidental removal in pediatric patients. Over a 4-year period, 62 children underwent EVD placement using this technique, with no cases of dislodgement, migration, or dislocation recorded. These findings are especially noteworthy given the well-documented challenges associated with maintaining the EVD position, particularly in children, who are at higher risk due to agitation, movement, and thinner scalp tissues.

EVD dislodgement remains among the most frequently reported complications in neurosurgical practice, contributing to increased morbidity, emergency re-interventions, prolonged hospitalization, and elevated healthcare costs [10-13]. The absence of dislodgement events in our study strongly supports the hypothesis that the two-step fixation approach, utilizing an N-shaped primary anchoring suture followed by a tension-resistant non-absorbable suture, provides excellent mechanical stability.

Compared with previously reported methods, such as single-suture fixation, staple-based techniques, or adhesive anchor systems, our approach offers several key advantages: it is composed solely of standard surgical materials, requires no specialized tools, and can be performed easily by new personnel. While mechanical fixation systems may demonstrate higher tensile strength in vitro [4,7,8], they may not be accessible in all healthcare environments and often entail additional cost and training. Our technique, in contrast, avoids reliance on additional commercial devices, adhesives, or staples, making it cost-effective and feasible across both high- and low-resource clinical settings. Therefore, the reproducibility and easy adaptability of our method render it particularly attractive for widespread adoption. Furthermore, the dual-functionality of the second non-absorbable suture, providing both secure catheter fixation and facilitating closure of the exit point following EVD removal, represents a practical advantage by streamlining the procedure and minimizing patient discomfort, particularly in the pediatric population.

Complications related to the fixation technique were, in this study, minimal and easily manageable. In three cases, the secondary non-absorbable suture was inadvertently cut during EVD removal, necessitating simple bedside closure of the EVD exit point, a task for which the suture was originally intended and is routinely performed in all other described techniques. In one patient, overly tight application of the primary suture impaired cerebrospinal fluid drainage, prompting a bedside re-fixation. Importantly, none of these events led to long-term sequelae or required operative intervention.

Despite these promising results, several limitations merit consideration. This study is retrospective and conducted at a single tertiary care center, which may limit the generalizability of findings. Additionally, while no significant difficulties in application were observed, the learning curve and procedural time required for this method were not formally evaluated and should be addressed in future prospective multicentre studies.

## Conclusions

This study presents a novel, easy, safe, and cost-effective suturing technique for securing EVDs in all patients, but especially in pediatric patients. The method demonstrated excellent catheter stability with no cases of dislodgement or infection across a diverse cohort, including patients undergoing surgery for posterior fossa tumors, intraventricular hemorrhage, and other intracranial pathologies. The simplicity, reproducibility, and independence from commercial fixation devices make this technique particularly well-suited for routine use in both high- and low-resource clinical settings. In addition, the dual-purpose nature of the second non-absorbable suture, serving as both fixation and post-removal closure, offers a practical advantage that reduces patient discomfort and procedural steps.

Further prospective, multicenter studies with larger sample sizes and expanded outcome measures are warranted to validate these findings and to potentially establish this technique as a standardized approach in neurosurgical practice.
